# RGS-YOLO: A lightweight solution for surface defect detection in wind turbines

**DOI:** 10.1371/journal.pone.0343966

**Published:** 2026-03-17

**Authors:** Zhiyuan Sun, Xu Wang

**Affiliations:** College of Electrical and Power Engineering, Hohai University, Nanjing, China; Guangdong University of Petrochemical Technology, CHINA

## Abstract

The defects in wind turbines not only affect energy generation efficiency but can also lead to significant damage if not repaired promptly. To address the challenges of low detection efficiency and high costs in the real-world industrial scenario of wind turbine defect detection, we have designed a lightweight detection model. First, this study introduces Receptive-Field Attention Convolution(RFAConv) and develops the Cross Stage Partial with 2 convolutions and feature fusion-Receptive-Field Attention Convolution(C2f-RFAConv) module, integrating it into the backbone network. This approach allows the model to focus on spatial features while accurately capturing local information in each region through its receptive field, significantly enhancing its feature extraction capabilities. Additionally, we incorporate Group Shuffle Convolution(GSConv) in the neck network to ensure that the model remains lightweight while maintaining a high level of accuracy. In the design of the detection head, we leverage the low redundant computation capability of Spatial and Channel reconstruction Convolution(SCConv), along with its ability to promote the learning of representative features, to develop a detection head-SCConv Head-that integrates classification and detection with low computational cost and parameters. All experimental results are reported as the average of no fewer than three independent runs to ensure the stability and reliability of the results. Experimental results show that, compared to the original You Only Look Once version 8 nano(YOLOv8n), our model reduces its size by 1.16 MB and decreases the floating-point operations by 3.5 G while improving the mean Average Precision (mAP) by 3.7%. These results demonstrate the effectiveness of our model in achieving lightweight performance.

## Introduction

With the increasing issues of energy shortages and environmental pollution, the development and utilization of wind power are regarded as key solutions to alleviate the energy crisis and promote green, low-carbon development. Effective utilization of wind energy is crucial to achieving sustainable development. Wind turbines, as the core equipment of wind power systems, directly impact the power generation efficiency and safe operation of wind farms. As wind turbines grow in size and operate in more complex environments, various surface defects may arise, such as fatigue cracks, corrosion, and wear. These defects not only decrease the overall efficiency of the turbines but also increase maintenance costs and may lead to equipment downtime, thereby affecting the stability of the power grid. Consequently, comprehensive and precise defect detection and timely maintenance of turbines are particularly important. This effort not only helps extend the lifespan of the equipment and improve power generation efficiency but also ensures the safety and economic viability of wind farms, providing strong support for the sustainable development of renewable energy.

In recent years, the demand for green energy has led to a rapid increase in the number and distribution of wind turbines. These turbines are often installed in harsh environments with high wind speeds, exposing their blades and structures to extreme conditions like strong winds, ice, and corrosion. These challenges not only reduce their performance but also shorten their lifespan [1]. Therefore, timely detection and resolution of faults and defects in wind turbines are critical [2]. Effective inspection and maintenance can significantly improve energy generation efficiency, ensuring stable operation under extreme conditions.

Currently, surface defect detection of wind turbines primarily relies on manual inspection. The existing methods can be broadly categorized into two types: traditional techniques such as infrared thermography [3], ultrasonic testing [4], and vibration analysis [5], which identify internal defects and structural anomalies based on physical principles; and drone-based visible light imaging, where drones capture video and human analysis is used to identify defects. While this aerial method improves detection efficiency and coverage, it still depends on manual interpretation, which introduces subjectivity and errors. Therefore, the introduction of more efficient deep learning object detection algorithms can enable more accurate defect identification.

Compared to traditional detection methods, deep learning technology offers a simpler and faster approach to object detection, typically without the need for extensive algorithmic support. Convolutional Neural Networks (CNNs) [6,7] are widely applied in the field of industrial inspection and can be categorized into two main types: two-stage detection and one-stage detection [8]. Representative algorithms for two-stage detectors include Faster R-CNN [9] and Mask R-CNN [10]. While two-stage object detection models excel in accuracy, they are relatively slow and require substantial computational resources, resulting in lower efficiency. To address these issues, researchers have proposed one-stage object detection models that do not require a candidate region generation phase and can directly produce probabilities for target classes and location coordinates. They achieve faster detection speeds by providing final detection results in a single pass. For example, one-stage detection algorithms such as the Single Shot MultiBox Detector (SSD) [11] and the You Only Look Once (YOLO) [12–16] series have gained significant popularity. The YOLO series algorithms enable rapid detection by simultaneously performing object recognition and bounding box regression, resulting in compact models and flexible deployment, making them widely adopted in the industrial sector.

Wang et al. [17] proposed a crack detector that utilizes Haar features to identify defect regions, enabling automatic detection of cracks in turbine blades. Sahir Moreno et al. [18] presented a concept for a robotic system that captures images of turbine blades for damage detection, along with a convolution-based visual inspection method for detecting surface damage. Yao et al. [19] designed an efficient detection algorithm based on YOLOX, focusing on identifying damage to wind turbine blades. This model enhances a lightweight backbone feature extraction network based on the Re-parameterized Visual Geometry Group Network(RepVGG) architecture to improve inference speed and incorporates a cascading feature fusion module, thereby increasing the sensitivity to features in small target areas and enhancing the detection capability for multi-scale target damage. Additionally, Zhang et al. [20] explored the application of the YOLOv5 algorithm for detecting damage in wind turbine blades. Their experimental results demonstrated that the model is capable of predicting the location and type of blade damage with accuracy close to human levels, highlighting the significance of image enhancement techniques for smaller training datasets. Martin Stokkeland et al. [21] introduced a visual module that enables a drone to independently calculate its distance from wind turbine blades and to use the Hough transform algorithm for blade detection. Ye et al. [22] proposed a Dual-Patch Lightweight Neural Network (DPLDN) for image dehazing and enhancement. Compared to existing technologies, this method demonstrates superior performance in the task of wind turbine blade image segmentation, showing potential for practical applications. Qiu et al. [23] proposed an improved YOLO network for detecting surface cracks in blades. This network enhances the detection capability for small targets, although it pays less attention to the detection of lighter-colored and weak-featured objects. While computer vision faces challenges in detecting internal damage and relies to some extent on large datasets, it excels in surface damage detection and demonstrates strong generalization capabilities in complex backgrounds. Therefore, employing computer vision methods can effectively improve surface defect detection, reduce downtime, and enhance wind energy efficiency. Yang et al. [24] introduced a deep learning model for blade defect detection that combines ensemble learning based on random forests with transfer learning. Initially, the Otsu method is employed to preprocess blade images, eliminating complex backgrounds, followed by integrating transfer learning and ensemble learning techniques into the detection task.

Currently, traditional detection methods such as infrared thermography, ultrasonic testing, and vibration analysis [25] face challenges related to high complexity and expensive costs. Although some existing algorithms for detecting wind turbine defects have achieved significant improvements in accuracy, they often focus solely on the detection performance of the models while neglecting the practical deployment of these models on hardware platforms. These algorithms typically involve substantial computational demands, making deployment difficult. Furthermore, the reliability of existing models is constrained by the limitations of turbine datasets. These challenges render current algorithms for surface defect detection in wind turbines inadequate in addressing the complexities and variabilities of real industrial scenarios. Therefore, innovative and more detailed solutions are urgently needed. To tackle these issues, we have designed a comprehensive dataset for the overall surface defects of wind turbines and proposed a lightweight high-performance surface defect detection algorithm based on YOLOv8n. Our algorithm aims to effectively address the aforementioned challenges and enhance the applicability and reliability of the model in practical applications. Our main contributions are summarized as follows:

1. We introduced a self-designed Cross Stage Partial with 2 convolutions and feature fusion-Receptive-Field Attention Convolution(C2f-RFAConv) module in the backbone network. This module integrates an attention mechanism and a cross-channel information fusion strategy, allowing the model to focus more effectively on the important areas of the input feature map, thereby improving its sensitivity to local feature differences.
2. In the neck network, we incorporated Group Shuffle Convolution(GSConv) convolution and the One-Shot Aggregation Network-based Ghost-Shuffle Cross Stage Partial module(VOV-GSCSP) module. The SConv convolution employs an efficient convolution strategy, optimizing the structure of the convolution kernels to enable more refined feature extraction, thereby enhancing the model’s detection capabilities in complex environments. Meanwhile, the VOV-GSCSP module improves feature fusion, increasing the model’s ability to capture multi-scale features while reducing computational load without compromising accuracy.
3. The original detection head of YOLOv8n is overly complex and not well-suited for industrial applications. Therefore, we combined it with Spatial and Channel reconstruction Convolution(SCConv) to design the SCConv Head, merging the classification and localization branches. This design maximizes detection accuracy while ensuring lightweight performance.

## Method

### The network architecture of YOLOv8n

In terms of the model backbone, YOLOv8n utilizes the CSPDarknet-53 architecture [14], which features a removal of one ConvModule layer. This architecture introduces a Cross-Stage Partial (CSP) network strategy that enhances the efficiency of feature information flow by dividing the feature map into two parts and incorporating cross-stage connections between them. The neck network combines a Feature Pyramid Network (FPN) [26] with a Pyramid Attention Network (PANet) [27], enabling efficient feature fusion through bidirectional interaction between shallow features passing to deeper layers and deep features being fed back to shallower layers. Additionally, the neck employs the C2f module, which effectively integrates channel and feature information to improve the model’s performance in complex tasks.

**Fig 1:**
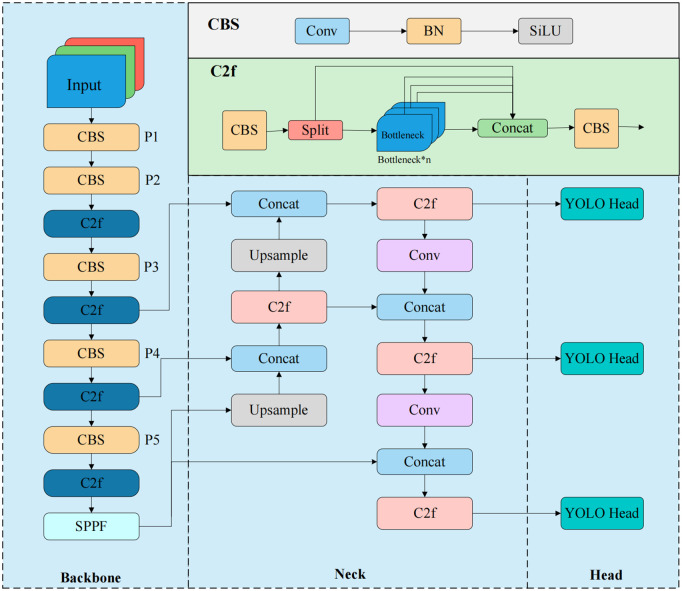
YOLOv8n network architecture. Backbone, which is responsible for feature extraction; Neck, which aggregates and fuses multi-scale features; and Head, which performs the final prediction.

In the detection head, YOLOv8n adopts a Decoupled-Head structure that separates the classification and localization tasks, each using different loss functions to optimize model performance. The localization task utilizes the Complete Intersection over Union (CIoU) loss, while the classification task employs Binary Cross-Entropy (BCE) loss to enhance classification accuracy. This design of the detection head contributes to improved detection precision for the model. The structure of YOLOv8n is illustrated in Fig 1.

### Improved network model

**Fig 2:**
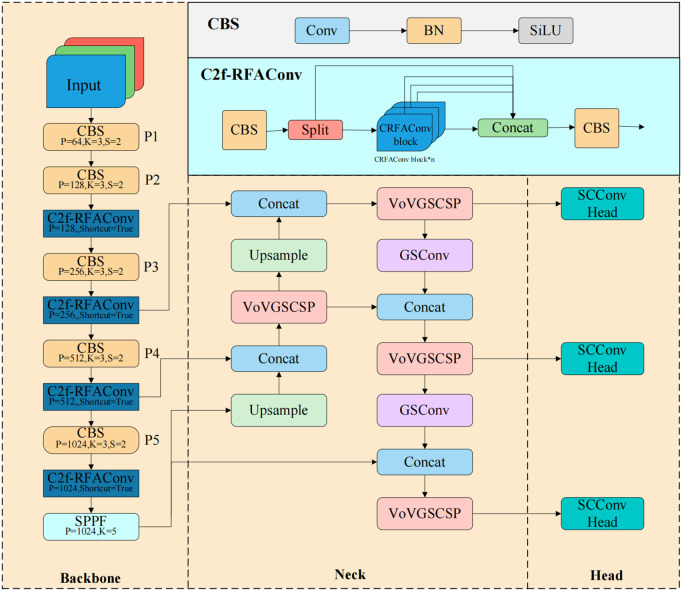
Improved network model. Compared with the baseline network, the backbone and neck are enhanced by incorporating GSConv and VoVGSCSP modules, while the C2f-RFAConv module is introduced to improve feature representation and information fusion efficiency.

Based on the needs of practical engineering applications, we propose a lightweight target detection model for wind turbines. This model significantly reduces the model size, the number of parameters, and the computational load, making it suitable for deployment on small hardware platforms. As shown in Fig 2, we initially replaced the C2f module in the model’s feature extraction component with the C2f-RFAConv module, which enhances the model’s sensitivity to local feature differences.

In the feature fusion layer, we employed GSConv and VOV-GSCSP to achieve a degree of lightweight design. The use of these two components also facilitates more reasonable and smoother feature fusion. In the detection head, we abandoned the decoupled head design in favor of merging the two branches, significantly improving the model’s detection speed and reducing its size. Additionally, we designed the SCConv Head detection head, which substantially decreases the model’s parameter count while minimally impacting accuracy, thus enhancing the detection speed of the model.

### C2f-RFAConv module

**Fig 3:**
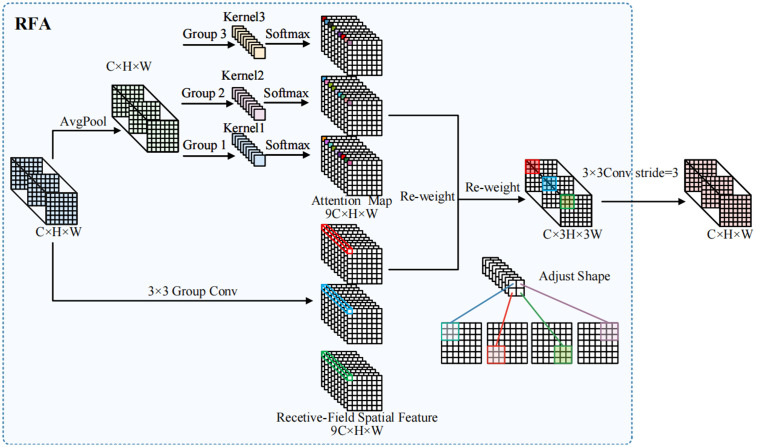
RFAConv structure. RFAConv integrates receptive-field spatial features to expand contextual perception and group convolution to improve computational efficiency and reduce parameter redundancy.


In the detection of wind turbine defects, turbines often operate in very complex environments, posing challenges to our high-performance detection capabilities. In the feature extraction component of YOLOv8n, a large number of traditional convolutions are employed. However, in traditional convolution operations, the parameters of the same convolution kernel are shared across different areas of the image, which hinders the model’s ability to capture local feature differences at various locations, ultimately impacting defect detection significantly. To address this issue, we introduced a novel convolution method called RFAConv [28], as illustrated in Fig 3. This approach employs a new convolution operation based on RFA, which integrates receptive field-aware spatial features with standard convolution kernels. This not only optimizes the parameter sharing issue of convolution kernels but also reduces computational overhead and improves training efficiency by replacing the traditional Unfold method with a fast Group Conv method. In general, the calculation of RFA can be expressed as:

$$F=Soft\max(g^{1\times 1}(AvgPool(X)))\times ReLU(Norm(g^{k\times k}(X)))=A_{rf}\times F_{rf}$$ (1)

Here, As shown in equations 1, *X* represents the input feature maps, k×k denotes the size of the convolution kernel, *Norm* stands for normalization, *F* is obtained by multiplying the attention map $A_{rf}$ with the transformed receptive-field spatial feature $F_{rf}$, and $g^{1\times 1}$ refers to the grouped convolution. Furthermore, RFAConv introduces a Receptive Field Attention (RFA) mechanism. Unlike traditional spatial attention mechanisms that focus solely on spatial features, RFA dynamically adjusts the convolution kernel parameters for each receptive field region, effectively addressing the parameter sharing problem associated with large convolution kernels and enhancing the network’s expressive power. Specifically, RFA assigns weights based on positional and contextual information to each receptive field region, allowing features at different locations to utilize distinct convolution kernel parameters, thereby capturing local differences more accurately.

**Fig 4:**
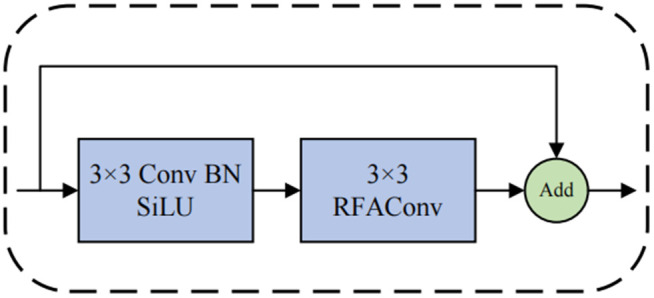
CRFAConv structure. The CRFAConv module integrates a CBS block with RFAConv, enabling effective feature extraction through receptive-field spatial features and efficient convolution operations.

**Fig 5:**
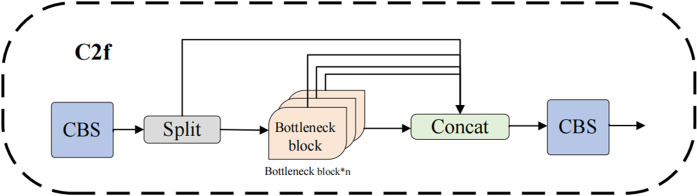
C2f structure. C2f is a feature fusion module employed in the neck of YOLOv8 to enhance information flow and multi-scale feature representation.

**Fig 6:**
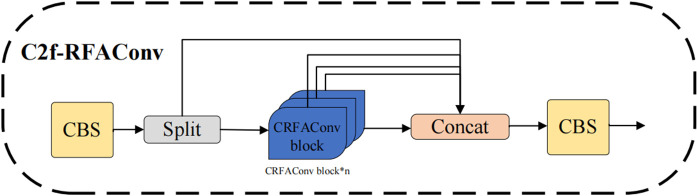
C2f-RFAConv structure. The original C2f module is enhanced by incorporating CRFAConv, aiming to strengthen feature representation while maintaining computational efficiency.

To address this issue, we replaced some of the traditional convolutions in the bottleneck of C2f with RFAConv convolutions, designing the CRFAConv module to enhance the capabilities of feature extraction and fusion, as illustrated in Fig 4. This enables the model to dynamically adjust convolution kernel parameters based on different receptive field regions, further improving the network’s feature representation and adaptability. In the backbone network of YOLOv8n, the C2f module establishes more connections through cross-layer feature fusion, allowing features to flow between different depths and thereby reducing information loss within deep networks. However, as shown in Fig 5, the extensive use of traditional convolutions for feature extraction within the C2f module somewhat weakens the model’s ability to capture local differences. In the C2f (cross-channel feature fusion) module, the input features are first processed through different convolutional or other feature transformation operations, followed by channel-wise weighted fusion. The attention-weighting mechanism in RFAConv interacts with this process: the attention-enhanced feature maps generated by RFAConv influence the cross-channel fusion process in the C2f module, particularly during the channel-weighted fusion stage. This interaction enables the fused features to become more representative and discriminative. Following this, we refined the C2f module to design the C2f-RFAConv module. C2f-RFAConv enhances the model’s feature extraction capabilities through channel-wise interactions and adaptive receptive fields, allowing for more accurate capture of target information. This leads to a significant improvement in reliability, especially in complex backgrounds, while generating richer and more diverse feature representations, as shown in Fig 6.

### Neck network based on GSConv and VoV-GSCSP

In the detection tasks for wind turbines, the complex and varying environments often significantly interfere with the capture of targets in images. If depthwise separable convolutions are directly employed for feature extraction within the network structure, a considerable amount of channel information can be lost, adversely affecting detection accuracy. To address this issue, we introduced a lightweight convolution structure—GSConv [29]—into the neck network.

**Fig 7:**
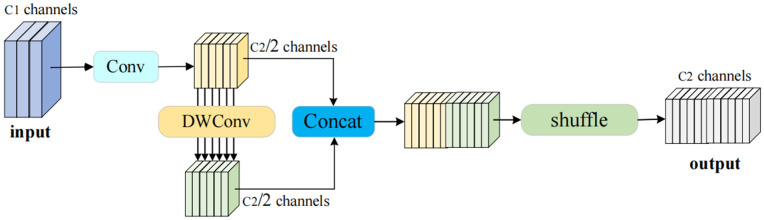
GSConv structure. GSConv consists of a depthwise convolution (DWConv) for lightweight spatial feature extraction and a channel shuffle operation to promote information exchange across channel groups.

GSConv represents an innovative hybrid convolution structure that seamlessly integrates standard convolution, depthwise separable convolution (DWConv), and Shuffle operations. This integration harnesses the strong feature extraction capabilities of standard convolution while capitalizing on the computational efficiency inherent in depthwise separable convolution. The operational framework of GSConv unfolds in a systematic manner, as illustrated in Fig 7. Initially, standard convolution is applied to the input features, facilitating downsampling and effectively compressing the spatial dimensions of the data. This step is crucial for reducing the computational burden in subsequent processes. Following the downsampling, DWConv is employed to extract nuanced spatial information that might be vital for understanding complex patterns within the data. The outputs generated from these two operations produce two distinct sets of features: channel-dense convolution (SC) and channel-sparse convolution (DSC). These feature sets are then concatenated along the channel dimension, which enhances the representation capability of the model by combining both rich and concise feature information.

Next, the Shuffle operation rearranges the channels of SC and DSC, allowing the high-density features from the standard convolution to be evenly distributed across the channels of the depthwise separable convolution. This facilitates thorough information fusion. This structure effectively mitigates the channel information loss problem inherent in depthwise separable convolutions by enabling uniform exchange of local features among different channels. At the same time, it significantly reduces computational overhead while enhancing inference efficiency without compromising feature representation capabilities.

As shown in equations (2)–(3), denotes the width of the input feature map, *H* denotes the height of the output feature map; $K_{1}\times K_{2}$ represents the size of the convolution kernel; $C_{1}$ is the number of input channels for each convolution kernel, and $C_{2}$ is the number of output channels of the feature map. Compared with depthwise separable convolution (DSC), the GSConv module exhibits lower computational cost and time complexity. Within the domain of wind turbine defect detection, this characteristic is particularly advantageous. The GSConv unit is capable of adaptively modifying both the size and shape of its kernels, enabling the network to capture fine-grained local structural patterns that are often associated with small or irregular defect regions. As a result, the module can selectively emphasize critical areas in the turbine components where cracks, corrosion, or surface wear are likely to occur. Such adaptability not only strengthens the sensitivity of the network to subtle defect cues but also enhances spatial localization accuracy.


$$Times_{DSC}=O(W\times H\times K_{1}\times K_{2}\times 1\times C_{2})$$ (2)

$$Times_{GSConv}=O[W\times H\times K_{1}\times K_{2}\times \frac{C_{2}}{2}\times (C_{1}+1)$$ (3)

**Fig 8:**
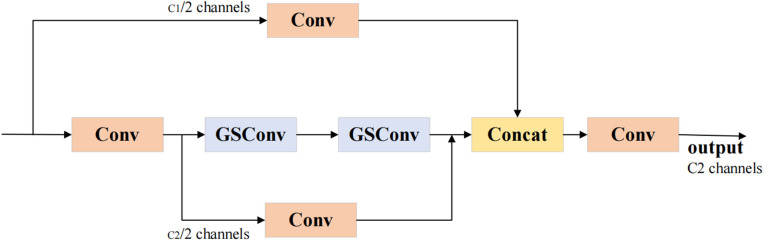
VOVGSCSP structure. The VoVGSCSP module incorporates GSConv to achieve efficient feature extraction.

Although GSConv excels at reducing computational complexity, there remains a need to introduce more efficient structures to further shorten inference time while maintaining accuracy. To this end, we designed a lightweight feature fusion module based on GSConv called VoV-GSCSP, inspired by the single-aggregation concept from VoVNet and the cross-stage partitioning strategy of CSPNet (as illustrated in Fig 8). This module functions similarly to C2f but incurs lower computational overhead.

In our proposed algorithm, we innovatively substituted all standard convolutions in the feature fusion network with GSConv layers, and we replaced the C2f module in the neck network with the VoV-GSCSP architecture. This approach not only preserves a rich capacity for feature representation but also enhances the model’s robustness when dealing with complex scenes and multi-scale targets. By implementing these changes, we effectively mitigate the decline in localization accuracy typically associated with deeper neural networks. Our results demonstrate a significant dual improvement in both computational efficiency and inference speed, achieved without compromising accuracy.

### SCConv head

**Fig 9:**
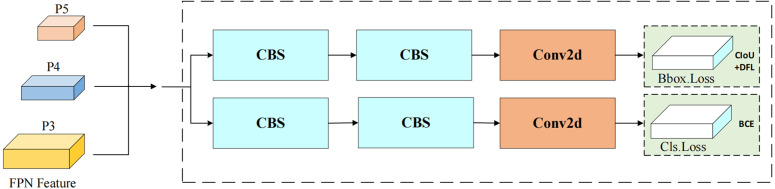
YOLOv8n detection head structure. The detection head adopts a decoupled design to separately perform bounding box regression and classification on multi-scale feature maps.

As shown in Fig 9, the decoupled head of YOLOv8n begins the feature extraction process by inputting feature maps P3, P4, and P5 from different layers into the detection head. The model then separates the classification task from the localization task, forming two independent branches: one branch focuses on classification to predict the object’s category, while the other branch is responsible for localization, primarily predicting the object’s position and size. This design approach aims to reduce interference between tasks, ensuring that classification and localization can be optimized independently within their respective branches.

However, this straightforward mechanical separation does not fully account for the feature interactions between classification and localization. In object detection tasks, classification and localization are complementary; ideally, their features should somewhat intermingle and influence each other. There exists a certain overlap and correlation between the semantic information needed for the classification branch and the spatial information required for the localization branch. An overly simplistic separation may result in the loss of important information, consequently affecting the model’s overall performance. The rich contextual information and details in the feature maps may not be effectively utilized during the transfer process, leading to constrained feature expressiveness in each branch.

**Fig 10:**
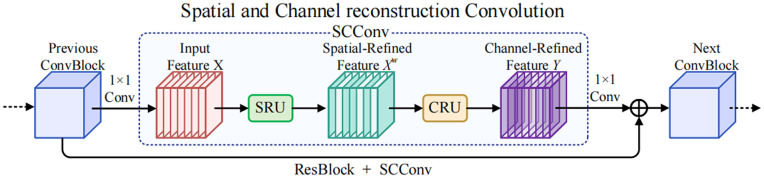
SCConv structure. The SCConv module is composed of a Spatial Reconstruction Unit (SRU) and a Channel Reconstruction Unit (CRU), which collaboratively enhance spatial and channel-wise feature representation.


To address the aforementioned issues and considering that in practical wind turbine defect detection scenarios, limited-resource drone devices are often used, the original parallel design of the YOLOv8n detection head leads to an excessive number of parameters, negatively impacting performance. Although CNNs have achieved remarkable performance in various computer vision tasks [30–32], this often comes at the cost of substantial computational resources, partly due to the redundancy in feature extraction by convolutional layers. In response, we employed an efficient convolution module, SCConv (Spatial and Channel Reconstruction Convolution) [33], as shown in Fig 10. SCConv aims to reduce redundant computations while enhancing the extraction of representative features in convolutional neural networks. Comprising two main components—Spatial Reconstruction Unit (SRU) and Channel Reconstruction Unit (CRU)—SCConv effectively mitigates spatial redundancy through a separable reconstruction approach and addresses channel redundancy via a separable transformation fusion strategy. Notably, SCConv is a plug-and-play architectural unit compatible with various convolutional neural networks, enabling straightforward replacement of standard convolutions. This flexibility allows for improved model performance without extensive modifications. By minimizing the computational overhead associated with redundant operations, SCConv contributes to faster training times and greater operational efficiency, making it especially valuable in resource-constrained large-scale applications. The parameters of the proposed SCConv module consist of:

$$P_{sc}=1\times 1\times \alpha C_{1}\times \frac{\alpha C_{1}}{r}+k\times k\times \frac{\alpha C_{1}}{gr}\times \frac{C_{2}}{g}\times g\;+1\times 1\times \frac{\alpha C_{1}}{r}\times C_{2}+(1-\alpha )C_{1}\times \frac{(1-\alpha )C_{1}}{r}\;+1\times 1\times \frac{(1-\alpha )C_{1}}{r}\times \left(C_{2}-\frac{1-\alpha }{r}C_{1}\right)$$ (4)

**Fig 11:**
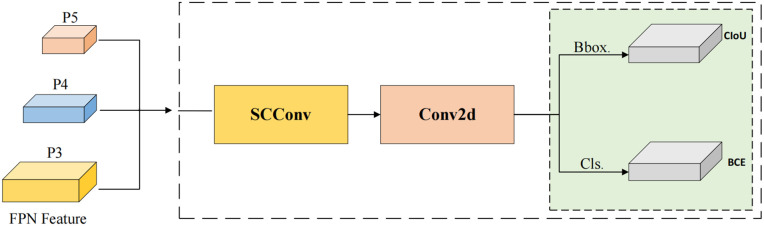
SCConv Head structure. SCConv is embedded into the coupled detection head to refine shared features for both classification and localization through spatial and channel reconstruction.

As shown in equations 4, *α* denotes the split ratio, *r* represents the squeeze ratio, *g* indicates the group size of the GWC operation, and $C_{1}$ and $C_{2}$ refer to the numbers of input and output feature channels, respectively. Building on this, we implemented lightweight improvements to the YOLOv8n detection head, designing the SCConv Head, as illustrated in Fig 11. We first merged the classification and localization tasks in the original detection head to allow these two tasks to operate in parallel. This design not only promotes information exchange between classification and localization, enhancing target detection accuracy, but also significantly reduces the model’s computational load and parameter count, thereby minimizing detection latency. This efficient design accommodates the resource limitations of devices such as drones, enabling faster and more effective defect detection in practical applications. Subsequently, we introduced SCConv, leveraging the combined effects of the SRU and CRU to effectively reduce redundant feature extraction in convolutional neural networks while enhancing feature expressiveness.

## Dataset

Given the extreme scarcity of existing datasets for wind turbine defects, there are virtually no publicly available and reliable data resources. Therefore, we conducted a systematic field data collection effort. Ultimately, we collected a total of 862 images of wind turbine defects, each with a resolution of 1280×1080. The dataset used in this study was independently collected by the authors, and the entire data collection and analysis process strictly adhered to institutional guidelines and ethical standards, ensuring compliance with privacy and data-sharing regulations. Subsequently, we used the LabelImg tool to annotate all the images, generating .txt label files suitable for YOLO. Based on the common types and characteristic manifestations of surface damage occurring during the operation of wind turbines, the defects were classified into five categories: leakage, paint, erosion, crack, and dirt, and the corresponding images were annotated accordingly. Among them, cracks are common damages generated on turbine blades due to inertia and vibration; if not repaired promptly, cracks may gradually propagate under alternating loads, eventually leading to fracture. Dirt is mainly caused by dust and other particles, irregular in shape and typically yellow-black in color; long-term accumulation may chemically react with rainwater, further evolving into other types of defects. Paint(Paint Peeling) usually occurs as a result of prolonged blade rotation, leading to the detachment of the protective coating, which—if left untreated—can cause further damage. Erosion refers to surface material degradation caused by the long-term effects of rain, oil contamination, and environmental exposure. Leakage manifests as oil seepage during extended turbine operation, usually dark in color and covering a relatively large area, with a clearly distinguishable appearance from cracks. Since some images contain multiple types of defects, statistics were collected based on the number of annotated bounding boxes: 641 leakage defects, 701 paint peeling defects, 732 dirt defects, 610 crack defects, and 672 corrosion defects. These defect images encompass not only the blades but also other parts such as the hub and tower, creating a more comprehensive dataset for wind turbine defects. Given the limited number of original images, we first divided a total of 862 original images into training, validation, and test sets in an 8:1:1 ratio. Then, we applied data augmentation techniques such as translation, cropping, rotation, and cloudy weather simulation to expand the training set to 2,522 images, and the validation and test sets to 315 images each.

Despite these efforts, this study still relies on a relatively small number of raw images, and the performance gains achieved may be partially influenced by extensive data augmentation. Although augmentation effectively increases data diversity and reduces overfitting, it may introduce distribution bias, as augmented samples are derived from the same underlying scenes and may not fully capture the variability encountered in real-world industrial environments. Consequently, the generalizability of the proposed method to wind farms with different turbine models, environmental conditions, or imaging setups may be limited. Future work will focus on collecting larger-scale, multi-site datasets and incorporating real-world operational data to further validate the robustness and industrial applicability of the proposed approach.

## Experimental setup and results analysis

### Evaluation criteria

To ensure the reliability and stability of our proposed model, all models were independently trained at least three times under identical experimental conditions, with the standard deviation across runs maintained within 0.3%. The model performance was subsequently evaluated using several widely adopted metrics, including Precision (P), Recall (R), and mean Average Precision at an Intersection over Union threshold of 0.5 (mAP@0.5). In addition, key statistical parameters such as True Positives (TP), False Positives (FP), and False Negatives (FN) were analyzed for a comprehensive assessment [34–36]. As shown in equations 5, TP denotes the true positives, representing defects correctly identified by the model, whereas FP refers to the false positives, indicating the number of normal samples incorrectly detected as defective. A higher precision value reflects the model‘s effectiveness in minimizing false positives and thus enhances the overall accuracy of defect detection.


$$\begin{array}{c} \begin{array}{cc} P & =\frac{TP}{TP+FP}\\ R & =\frac{TP}{TP+FN}\\ P_{AP} & =\int_{0}^{1}P(R)d_{R}\\ P_{mAP} & =\frac{\Sigma_{i=1}^{n}AP_{i}}{n} \end{array}\; \end{array}$$ (5)

### Ablation experiments

In this study, all experiments were conducted under a unified experimental environment to ensure the reliability and comparability of the results. The specific environment configurations and training parameters are presented in Table 1. In addition, to comprehensively evaluate the model performance, ablation experiments were conducted to verify the reliability of the proposed model.

**Table 1:** Training configuration and experimental settings.

Item	Setting
Programming Language	Python 3.8.16
Deep Learning Framework	PyTorch 2.0.0
CUDA Version	CUDA 11.8
Optimizer	SGD (Stochastic Gradient Descent)
Batch Size	4
Initial Learning Rate	0.01
Epochs	200
Learning Rate Strategy	Warm-up + Cosine Annealing

As shown in Table 2, we first compared Experiment B and Experiment C. The introduction of C2f-RFAConv allowed the model to extract differential features from local regions, enabling it to dynamically capture feature disparities. This resulted in improved defect detection performance for wind turbines with minimal additional computational load, leading to a 3.28% increase in mAP@0.5. When comparing Experiment B and Experiment D, the incorporation of GSConv and VOVGSCSP into the neck network, with its new mixed convolution structure, resulted in a decrease of 0.8G in the model’s floating-point operations while also reducing the model size by 0.37M. Furthermore, the adoption of this efficient structure increased the mAP@0.5 by 1.42%. In comparing Experiment B and Experiment E, our designed SCConv Head, which integrates classification and localization, led to a reduction of 2.9G in floating-point operations and a decrease of 0.91M in model size. However, while this fusion approach significantly reduced computational load and parameter count, it also caused a slight decline in accuracy. To mitigate this issue, we employed SCConv to enhance our model’s performance, resulting in only a 0.17% decrease in mAP@0.5, which is acceptable. Finally, when comparing the remaining experiments F, G, and H, we confirmed the reliability of our model’s results. Improvements made to the backbone, neck, and head networks of the model not only increased its lightweight capabilities but also further enhanced its performance.

**Table 2:** Comparison of our RGS-YOLO with the classic methods.

No	Method	mAP@0.5(%)	GFLOPs(G)	Model Weights size(MB)
A	Ours	91.89 ± 0.15	5.2	4.80
B	YOLOv8n	88.19 ± 0.20	8.7	5.96
C	+ C2f-RFAConv	91.47 ± 0.18	9.0	5.64
D	+ GSConv	89.61 ± 0.22	7.9	5.59
E	+ SCConv Head	88.02 ± 0.24	5.8	5.05
F	+ C2f-RFAConv + GSConv	91.95 ± 0.16	8.2	5.27
G	+ C2f-RFAConv + SCConv Head	91.21 ± 0.19	6.1	4.94
H	+ GSConv + SCConv Head	89.29 ± 0.23	5.0	4.92

### Detection performance of different models

To further evaluate the detection performance of our proposed model, we conducted comparative experiments with several mainstream models using our designed dataset. The experimental results are summarized in Table 3. Faster R-CNN, a two-stage algorithm, divides detection into two phases. Although it possesses a relatively high number of parameters and a large model size—with an mAP@0.5 of 77.34%—its considerable size is not conducive to real-time deployment. As a result, due to hardware performance limitations and cost, two-stage detection frameworks are seldom used in engineering applications. Among one-stage detectors, the SDD algorithm is a representative single-stage model. However, due to its poor feature extraction capabilities, it exhibits significant gaps in detection precision, recall rate, and mAP@0.5 compared to the YOLO series, achieving an mAP of only 74.86%. The YOLO series has been continuously improved as a representative one-stage detector. With its efficient and robust architecture, it demonstrates strong capabilities in small object detection and multi-class classification. YOLOv5, an earlier classic algorithm, offers reasonable detection performance with an mAP@0.5 of 82.97%. However, it is not the optimal model for today’s variable and complex environments. Our goal was to develop a lightweight detection model, so we included a newer lightweight algorithm, MobileNetV2, for comparison. We noted that MobileNetV2 has significantly fewer parameters and a model size of 6.79M compared to SDD and Faster R-CNN. Nonetheless, its feature extraction capabilities are relatively weak, particularly for extracting features from small targets, resulting in a model accuracy that is 8.5% lower than ours. Moreover, a latency comparison was conducted on desktop-class GPU hardware, and the results indicate that the proposed model achieves the smallest model size and the lowest inference latency among all benchmark models under this experimental setting. These results suggest promising potential for deployment on resource-constrained platforms; however, further validation on UAVs or edge devices is required to confirm its practical suitability.

**Table 3:** Comparison of ablation experimental results.

Method	mAP@0.5(%)	mAP@[0.5:0.95](%)	Prec(%)	Recall(%)	Params(M)	Latency(ms)
Faster-RCNN	77.34 ± 0.13	45.21 ± 0.21	80.67 ± 0.07	73.38 ± 0.24	58.74	95.4 ± 0.18
SSD	74.86 ± 0.28	41.73 ± 0.16	77.62 ± 0.11	70.79 ± 0.29	24.39	28.6 ± 0.20
MobileNetV3-SSD	78.83 ± 0.27	46.84 ± 0.21	79.19 ± 0.20	77.68 ± 0.24	5.12	12.6 ± 0.15
EfficientDet-D0	79.11 ± 0.09	49.78 ± 0.17	80.91 ± 0.25	77.13 ± 0.13	3.90	8.4 ± 0.14
YOLOv5n	82.97 ± 0.26	58.84 ± 0.14	84.51 ± 0.23	80.13 ± 0.12	5.04	5.7 ± 0.12
MobileNetV2-1.4	83.39 ± 0.19	63.92 ± 0.25	84.99 ± 0.22	81.75 ± 0.08	6.79	8.3 ± 0.13
YOLOv8n	88.19 ± 0.20	69.56 ± 0.21	89.46 ± 0.15	86.76 ± 0.10	5.96	6.9 ± 0.11
YOLOv11n	90.16 ± 0.18	72.93 ± 0.19	91.14 ± 0.20	89.43 ± 0.28	5.94	6.1 ± 0.10
Ours	91.89 ± 0.15	74.27 ± 0.16	95.71 ± 0.17	91.73 ± 0.15	4.80	4.7 ± 0.09

When comparing the remaining YOLO series algorithms, YOLOv8n and YOLOv11 show only minor differences, with roughly comparable detection efficiency. Our model, however, employs a novel approach that enhances its sensitivity to local differences while greatly reducing both parameter count and computational load. When compared to the latest YOLOv11, our model achieves a 1.73% increase in mAP@0.5, while also exhibiting a substantial reduction in model size. This evidences that our model outperforms existing models in terms of performance.

### Performance on the public dataset

To demonstrate the reliability of our model, we compared it with a public insulator dataset. Currently, there is no publicly available dataset specifically for wind turbine defects; however, the insulator dataset shares similarities with wind turbine defect datasets in terms of features: both involve high-altitude inspections conducted by drones and cover various types of defects. Therefore, we chose this public insulator dataset for our experiments. In this dataset, the resolution width of the image pixels ranges from 2000 to 5000, and the height ranges from 2000 to 3000. The dataset is constructed based on original images and mainly includes two types of defects: pollution flashover and damage. There are a total of 1600 image samples in the dataset, which is divided in a ratio of 7:2:1, where 0 represents the pollution flashover defect, 1 represents the damage defect, and 2 represents the insulator.

As shown in Table 4, ablation experiments were conducted on the public insulator dataset to verify that the proposed improvements in this paper are effective not only on our self-constructed wind turbine dataset but also in other related scenarios. The experimental results indicate that when all improved modules are integrated into the original YOLOv8n network, the model’s mAP@0.5 increases by 2.53% compared to the original YOLOv8n network without any improvements.It is noteworthy that when only the SCConv Head module is added to the original YOLOv8n network, the detection accuracy declines. This result is consistent with the ablation experiment results on the wind turbine dataset. Analysis shows that while the SCConv Head module achieves model lightweighting, it may weaken the capability to extract fine-grained features, leading to a slight decrease in detection performance. However, SCConv Head significantly reduces the computational cost by 2.9 GFLOPs and the model size by 0.9 MB. This loss in accuracy is acceptable, especially in practical applications where lightweight design and resource constraints are considered; such trade-offs are reasonable.

**Table 4:** Ablation experiments on the public dataset.

Method	C2f-RFAConv	GSConv	SCConv Head	mAP@0.5(%)	GFLOPs(G)	Model Weight Size(Mb)
	×	×	×	89.95 ± 0.25	8.7	5.97
	✓	×	×	91.82 ± 0.20	9.0	5.68
YOLOv8n	×	✓	×	91.01 ± 0.18	7.9	5.62
	×	×	✓	89.47 ± 0.28	5.8	5.07
	✓	✓	✓	92.48 ± 0.23	5.2	4.81

Table 5 presents a comparison of different models across various evaluation metrics. As shown in the table, compared to YOLOv8n, the model proposed in this paper improves the recall rate by 4.86% and enhances the mAP@0.5 metric by 2.53%. This indicates that the model demonstrates good detection performance across different scenarios. Although there is a slight decrease in overall accuracy compared to the results on the original wind turbine dataset, it is important to note that this model was specifically designed for the wind turbine domain. The types of defects it faces differ from those in the public insulator dataset. Thus, the model still achieves performance improvements on this public dataset, further validating its effectiveness and generalization ability.

**Table 5:** Model comparison experiments on the public dataset.

Method	Precision(%)	Recall(%)	mAP@0.5(%)	mAP@[0.5:0.95](%)
Faster-RCNN	89.56 ± 0.07	77.66 ± 0.14	83.16 ± 0.26	55.94 ± 0.22
MobileNetV2-1.4	86.35 ± 0.15	81.65 ± 0.23	84.90 ± 0.21	54.66 ± 0.20
YOLOv8n	91.58 ± 0.22	83.87 ± 0.19	89.95 ± 0.25	57.87 ± 0.24
YOLOv11n	92.27 ± 0.20	85.58 ± 0.11	90.79 ± 0.09	57.26 ± 0.22
Ours	95.71 ± 0.18	88.73 ± 0.17	92.48 ± 0.23	58.79 ± 0.19

### Empirical detection performance

**Fig 12:**
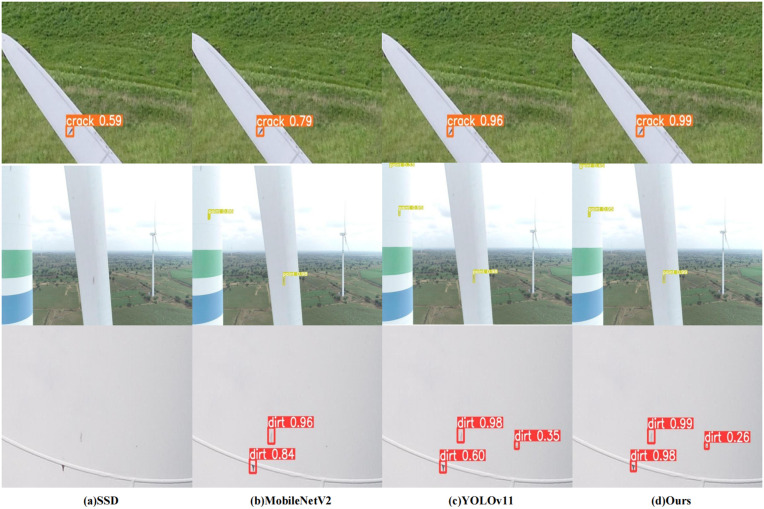
Detection results comparison. Each row represents a different input image, and each column corresponds to the detection results produced by a different model.

To validate the performance of our proposed wind turbine surface defect detection model in engineering application tasks, we used drones to simulate real-world conditions for image capture and dataset creation for comparison purposes. Our designed model not only has a small model size and significantly fewer parameters but also achieves the highest detection accuracy and reliability in identifying defect targets compared to other models. As shown in the first row of Fig 12, each model detected surface defects, but with considerable differences in detection accuracy. Our model demonstrated the best detection accuracy among the four models tested. In the second row, the defect targets were placed against a complex background, with multiple defects present, posing a significant challenge to the models‘ detection capabilities. The SSD model failed to detect any defects due to its weak feature extraction ability. Although MobileNetV2 identified two defect targets, its accuracy was not high, and it experienced missed detections because its model structure is relatively simplistic, with limited multi-scale feature extraction capabilities. Both our model and YOLOv11 detected all defect targets, but our model achieved the highest accuracy, further proving its performance. In the third row, the detection images featured tower structures, which are often white, highlighting defects due to contrast, although there may be numerous defect targets. The SSD and MobileNetV2 models failed to detect all defect targets, while both YOLOv11 and our model identified the targets. However, our model maintained the highest detection accuracy. This is attributed to the strong local feature extraction capability of the C2f-RFAConv module in our network, enabling it to effectively identify defects with strong contrast.

In summary, the improved model outperformed existing models in detecting wind turbine defect targets from drone-captured images, demonstrating superior accuracy and robustness. This indicates its strong potential for practical engineering applications. Our model exhibited reliability in detecting multiple targets under complex backgrounds, and being lightweight, it is well-suited for deployment on small hardware platforms.

## Conclusions

To address the issue of inadequate datasets for wind turbine defect detection, we designed a new, comprehensive surface defect dataset for wind turbines. To overcome the problem of existing models having excessively large parameter counts—which limits their performance on hardware—we proposed a novel lightweight algorithm for wind turbine defect detection. Compared to the original YOLOv8n model, we introduced a self-designed C2f-RAFConv module into the backbone network, significantly enhancing the model’s ability to extract local differential features. In the neck network, we incorporated GSConv and VOVGSCSP modules to improve the model’s feature fusion capability. Finally, we designed a new detection head that merges the originally separate branches (classification and localization) into a single branch, greatly reducing both computational load and parameter count. At the same time, the introduced SCConv module effectively ensured an improvement in model performance without causing any significant decline. Experimental results indicate that the improved model not only achieves high accuracy while maintaining a lightweight design but also has significant industrial application potential. This algorithm addresses the challenge of insufficient sample data for detecting surface defects on wind turbines, offering a rapid and efficient solution for defect identification. Although performance was measured on desktop GPU hardware, the lightweight nature of the model suggests it could be suitable for edge devices, such as UAVs, for real-time defect detection. However, further performance validation on UAVs or edge devices will be necessary before full deployment. Looking ahead, future work will focus on identifying smaller, more subtle defects on wind turbine surfaces. We aim to develop more sophisticated models that will significantly enhance the accuracy and overall performance of defect detection. Additionally, we will explore various deep learning techniques to further improve the model’s adaptability and robustness against varying environmental conditions. This future work will not only refine our detection methods but also contribute to advancing maintenance strategies for wind turbines, ultimately ensuring their operational efficiency and longevity.
